# Berberine enhances tumor necrosis factor-related apoptosis-inducing ligand-mediated apoptosis in breast cancer

**DOI:** 10.3892/ol.2013.1434

**Published:** 2013-07-01

**Authors:** ALAA REFAAT, SHERIF ABDELHAMED, HIDEO YAGITA, HIROKI INOUE, SATORU YOKOYAMA, YOSHIHIRO HAYAKAWA, IKUO SAIKI

**Affiliations:** 1 Division of Pathogenic Biochemistry, Department of Bioscience, Institute of Natural Medicine, University of Toyama, Toyama 930-0194, Japan; 2 Department of Immunology, Juntendo University School of Medicine, Tokyo 113-8421

**Keywords:** breast cancer, berberine, coptisine, TNF-related apoptosis-inducing ligand, apoptosis

## Abstract

Berberine (BBR) has been used for the treatment of bacterial and fungal infections and also for cancer-associated symptoms such as diarrhea. Furthermore, it has been reported that BBR may have direct antitumor effects. Although evidence supports the theory that tumor necrosis factor (TNF)-related apoptosis-inducing ligand (TRAIL) is a promising candidate for treating cancer, its usage may be limited due to the resistance to the TRAIL-induced apoptosis of cancer cells. In the present study, the effect of BBR on TRAIL-induced antitumor effects was investigated *in vitro* using recombinant TRAIL and *in vivo* using a 4T1 murine breast cancer model in combination with anti-DR5 (death-inducing TRAIL receptor) monoclonal antibody therapy. BBR sensitized human breast cancer cell lines to TRAIL-mediated apoptosis *in vitro*. The combination of BBR and recombinant TRAIL significantly activated caspase-3 and PARP cleavage in TRAIL-resistant MDA-MB-468 cells. Furthermore, BBR in combination with TRAIL more effectively induced apoptosis compared with coptisine (COP), which is structurally related to BBR. In a murine 4T1 breast cancer model, BBR treatment enhanced the efficacy of anti-DR5 antibody therapy against primary tumor growth and lung metastasis. Thus, BBR may become a new adjuvant for overcoming the resistance of cancer cells to TRAIL/DR5-mediated therapy.

## Introduction

Breast cancer is the most common malignancy in females and the second leading cause of cancer-related mortality in developed countries (1,2). Despite advances in the early detection and treatment of breast cancer, mortality remains significant due to the invasiveness and acquired resistance to current therapies (3). Thus, it is extremely important to identify new adjuvant therapies that sensitize breast cancer cells to current treatments and reduce possible side-effects.

Tumor necrosis factor (TNF)-related apoptosis-inducing ligand (TRAIL) is known to be a potential agent for anticancer treatment. TRAIL induces apoptosis by binding to its death-inducing receptors, DR4 and DR5, on cancer cells (4). Although TRAIL is known to selectively induce apoptosis in cancer cells (5,6), the development of partial or complete resistance is a potential limitation of TRAIL treatment. Several studies have indicated certain potential candidates that may be used in combination with TRAIL for overcoming TRAIL resistance in cancer cells (7–9)

Berberine (BBR) and its structurally related compound coptisine (COP) are isoquinoline alkaloids that occur in the rhizome of a number of valuable medicinal plants, including *Coptis chinensis* and *Coptis japonica*(10). Previous studies have demonstrated that *Coptis* extracts show anticancer activities (11–13). Notably, BBR has been extensively studied for its cytotoxic activities against several human cancer cells by inducing cell cycle arrest, apoptosis and/or inhibiting cell migration and invasion (14–21). Regardless of the similarities of their structures, COP is known to exhibit a limited number of biological activities compared with BBR. Notably, BBR has been shown to exert anticancer effects without affecting normal cells (22,23), indicating that BBR is an attractive anticancer compound (24). Although BBR sensitizes and induces TRAIL-mediated apoptosis in human renal cancer cells by downregulating c-FLIP and Mcl-1 (25), little is known with regard to the therapeutic potential of BBR and its associated compounds in combination with TRAIL.

In the present study, the synergistic effects of either BBR or COP were studied in combination with recombinant TRAIL in TRAIL-sensitive (MDA-MB-231) and -resistant (MDA-MB-468) human breast cancer cells. The therapeutic efficacy of BBR and anti-DR5 antibody in combination was further demonstrated in a murine 4T1 breast cancer model. The present study aimed to demonstrate that BBR may be a new adjuvant for overcoming the resistance of cancer cells to TRAIL/DR5-mediated therapy.

## Materials and methods

### Antibodies and reagents

Phospho-specific antibodies against p53 (Ser-15) and p65 (Ser-536), in addition to PARP-1, caspase-3 and Mcl-1, were purchased from Cell Signaling Technology (Danvers, MA, USA). The antibody against β-actin (C-11) was obtained from Santa Cruz Biotechnology, Inc. (Santa Cruz, CA, USA). Recombinant human TRAIL Apo II ligand (rTRAIL) was obtained from PeproTech, Inc. (Rocky Hill, NJ, USA). Chrompure Syrian hamster IgG was purchased from Jackson ImmunoResearch Laboratories, Inc (West Grove, PA, USA). BBR chloride (for *in vitro* experiments) and BBR chloride n-hydrate (for *in vivo* experiments) were purchased from Wako Pure Chemical Industries, Ltd. (Osaka, Japan). COP chloride was supplied by the medicinal plant library of the Institute of Natural Medicine (University of Toyama, Japan). BBR chloride and COP chloride were dissolved in dimethylsulfoxide. An agonistic anti-DR5 monoclonal antibody (MD5-1) was prepared as described previously (26).

### Cell culture

MDA-MB-231, MDA-MB-468 and Panc-1 cells were maintained in Dulbecco's modified Eagle's medium (DMEM) supplemented with 10% fetal calf serum, 2 mM glutamine, 100 U/ml penicillin and 100 μg/ml streptomycin at 37°C in 5% CO_2_. 4T1 cells were maintained in RPMI-1640 medium supplemented with 10% fetal calf serum, 2 mM glutamine, 100 U/ml penicillin and 100 μg/ml streptomycin at 37°C in 5% CO_2_.

### Cell viability assay

The viability of the cells was assessed using a WST-1 Cell Counting kit (Wako Pure Chemical Industries, Ltd.). Briefly, cell suspensions in DMEM containing 10% FBS were seeded into a 96-well plate (5–8×10^3^/80 μl/well) and incubated at 37°C. Subsequent to a 24-h incubation period, an additional 10 μl medium containing the test compounds at various concentrations was added to the wells. After 30 min, 10 μl medium containing rTRAIL was added, followed by further incubation for 24 h. WST-1 solution (10 μl) was added to each well 2 h prior to the end of the experiment. The absorbance at 450 nm was measured using a microplate reader. Cell viability was determined from the absorbance of soluble formazan dye generated by the living cells.

### Immunoblotting

Following stimulation, whole-cell lysates were prepared as described previously (27). The cell lysates were resolved by 7.5, 10, 12.5 or 15% sodium dodecyl sulfate-polyacrylamide gel electrophoresis and transferred to an Immobilon-P nylon membrane (Millipore, Billerica, MA, USA). The membrane was probed with each primary antibody.

### Animal model and treatment

Female, six-week-old BALB/c mice were purchased from Japan SLC Inc. (Hamamatsu, Japan). The mice were maintained in the Laboratory for Animal Experiments, Institute of Natural Medicine, University of Toyama (Toyama, Japan). Food and water were freely available. The study was conducted in accordance with the standards established by the Guidelines for the Care and Use of Laboratory Animals and approved by the ethics committee of the University of Toyama. The 4T1 cells were harvested and resuspended in cold phosphate-buffered saline (PBS). The cell suspensions (5×10^5^/50 μl/mouse) were injected into the mammary fat pads of the anesthetized mice. Five mice in each group received BBR in 0.5% carboxymethyl cellulose solution (100 mg/kg/day) or vehicle by oral administration six times a week, starting one day after tumor implantation. The volume of the tumors was tracked twice weekly starting from day 10, and the weight was eventually obtained on day 16 following removal of the primary tumor from the implantation site. The agonistic anti-DR5 antibody (MD5-1), was injected i.p. (10 μg/mouse) on days 8 and 13 in the designated groups. The primary tumor size was measured using a caliper square along the long axis (a) and short axis (b), and the tumor volume was calculated by the following formula: Tumor volume (mm^3^) = ab^2^/2. Subsequent to sacrificing the mice on day 31, Bouin's solution was infused into the lungs and the anti-tumor effect was evaluated by counting the number of metastasized tumor colonies on the surface of the lungs.

### Statistical analysis

Data are presented as the mean ± SEM of n experiments as indicated. The statistical analysis was performed using JMP software (version 10; SAS Institute Inc., Cary, NC, USA). The statistical significance between conditions was determined using a pairwise comparison applying the Tukey-Kramer honestly significant difference (HSD) method. P<0.05 was considered to indicate a statistically significant difference.

## Results

### BBR sensitizes TRAIL-sensitive and -resistant human breast cancer cells to TRAIL-induced apoptosis

**Figure 1:**
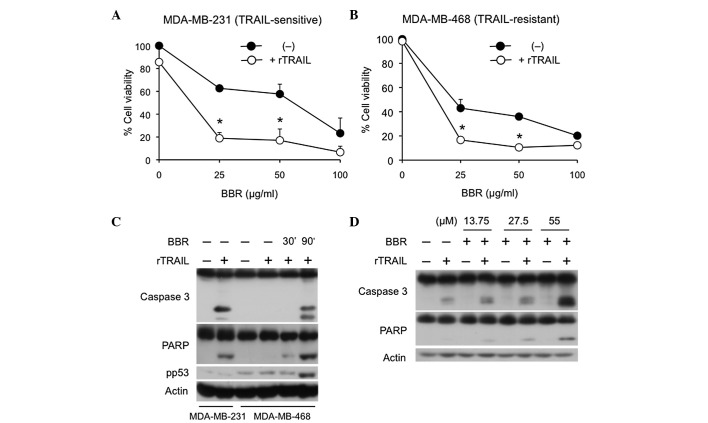
BBR sensitizes TRAIL-induced apoptotic cell death in human breast cancer cells. (A) TRAIL-sensitive MDA-MB-231 or (B) TRAIL-resistant MDA-MB-468 were pre-incubated with various concentrations of BBR for 30 min and further incubated in the presence or absence of rTRAIL (50 ng/ml) for 24 h. Absorbance was measured following incubation with WST-1 reagent and cell viability was calculated as a percentage compared with the untreated control cells. Data are expressed as the mean ± SEM of at least three triplicates of each experiment and significant differences between groups are shown as ^*^P<0.05. (C) MDA-MB-468 cells were pretreated with BBR (135 μM) for 30 or 90 min, followed by incubation in the presence of TRAIL (50 ng/ml) for 3 h. Whole cell lysates were analyzed by immunoblotting for the indicated proteins. (D) MDA-MB-468 cells were pretreated with BBR (13.7, 27.5 or 55 μM) for 90 min, followed by incubation in the presence or absence of TRAIL (50 ng/ml) for 3 h. Whole cell lysates were analyzed by immunoblotting for the indicated proteins. TRAIL, TNF-related apoptosis-inducing ligand; BBR, berberine; pp53, phosphorylated p53.

To evaluate the direct cytotoxic activity of BBR and BBR combined with TRAIL on the breast cancer cells, TRAIL-sensitive (MDA-MB-231) and -resistant (MDA-MB-468) human breast cancer cell lines were selected. Although BBR treatment alone caused only a marginal decrease in MDA-MB-231 cell viability, the combination of rTRAIL and BBR resulted in a significant synergistic effect in reducing the cell viability of the TRAIL-sensitive MDA-MB-231 cells (Fig. 1A). Notably, the MDA-MB-468 cells were relatively more sensitive to BBR treatment compared with the MDA-MB-231 cells, and furthermore, the combination of rTRAIL and BBR showed a synergistic effect even in the TRAIL-resistant MDA-MB-468 cells (Fig. 1B). These results clearly indicated that BBR not only synergized with TRAIL, but that it also sensitized the TRAIL-resistant cancer cells to TRAIL. To determine the kinetics of sensitization by the combination of TRAIL and BBR, the MDA-MB-468 cells were treated with rTRAIL and BBR and then the caspase-3 and PARP cleavage levels were measured. As shown in Fig. 1C, the cleavage of caspase-3 and PARP was induced by the treatment with a combination of rTRAIL and BBR for 90 min. The activation of the p53 tumor suppressor protein was also observed (Fig. 1C). By testing the treatment with various doses of BBR in combination with rTRAIL, it was observed that 55 μM BBR induced significant cleavage of caspase-3 and PARP (Fig. 1D). Such induction of the apoptotic pathway was also observed in another TRAIL-resistant human pancreatic cancer cell line Panc-1 (data not shown), suggesting that the sensitizing effect of BBR to TRAIL-induced apoptosis is not restricted to MDA-MB-468 breast cancer cells.

### BBR is more effective than COP in sensitizing the cells to TRAIL-induced apoptosis

**Figure 2:**
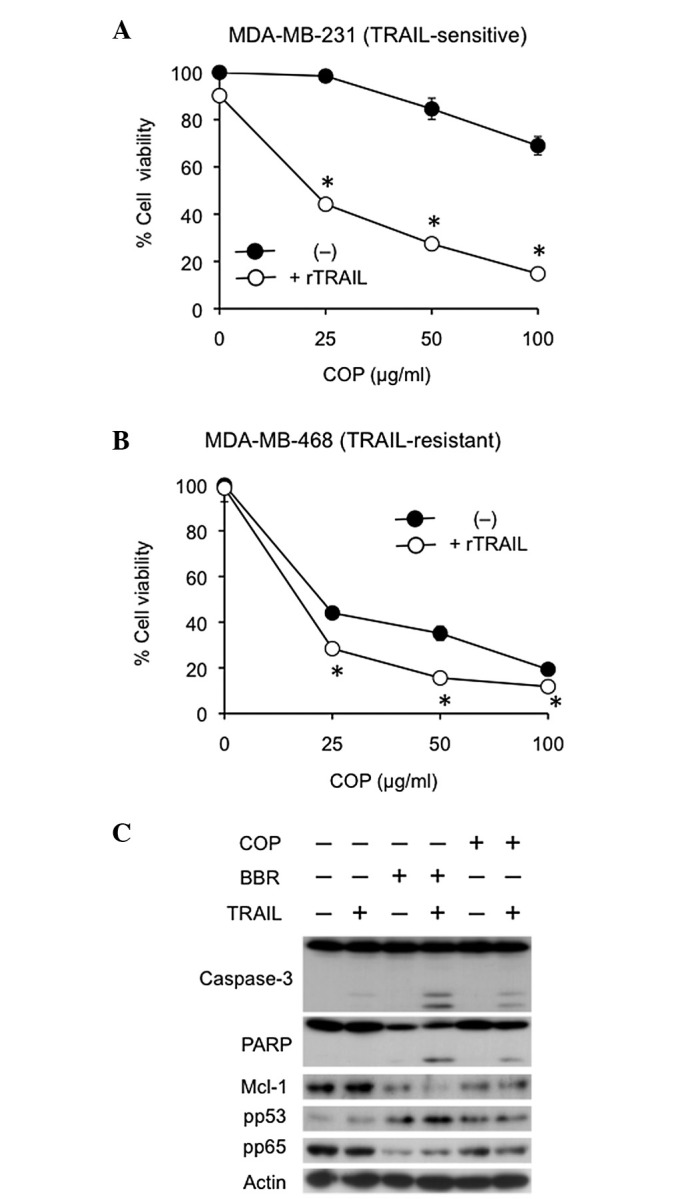
Efficacy of BBR and COP in sensitizing the cells to TRAIL-induced apoptosis. (A) MDA-MB-231 or (B) MDA-MB-468 cells were pre-incubated with various concentrations of COP for 30 min and further incubated in the presence or absence of TRAIL (50 ng/ml) for 24 h. Absorbance was measured following incubation with WST-1 reagent and cell viability was calculated as a percentage compared with untreated control cells. Data are expressed as the mean ± SEM of at least three triplicate experiments and significant differences between groups are shown as ^*^P<0.05. (C) MDA-MB-468 cells were pretreated with BBR or COP (55 μM) for 90 min, followed by incubation in the presence or absence of TRAIL (50 ng/ml) for 3 h. Whole cell lysates were analyzed by immunoblotting for the indicated proteins. TRAIL, TNF-related apoptosis-inducing ligand; BBR, berberine; COP, coptisine; pp53, phosphorylated p53; pp65, phosphorylated p65.

It is known that BBR and COP are structurally related compounds that occur in a variety of medicinally important herbal plants. Since the effective combination of BBR with TRAIL-induced apoptosis had been observed, the potential effect of COP in sensitizing the cells to TRAIL-induced apoptosis was further investigated. As shown in Fig. 2A and B, a similar effect of COP was observed in the induction of cytotoxicity against the MDA-MB-468 cells to BBR. To further directly compare the efficacy of BBR and COP in the sensitization of TRAIL-induced apoptosis, the activation of the apoptotic pathway was studied using rTRAIL treatment in combination with either BBR or COP. As shown in Fig. 2C, BBR showed a more prominent effect on the sensitization of the cells to TRAIL treatment in the MDA-MB-468 cells, as determined by the cleavage of caspase-3 and PARP. In addition, the inhibition of Mcl-1 and phosphorylated-p65 and the induction of phosphorylated-p53 were observed following the combined treatment with rTRAIL and BBR or COP, which was also reported in previous studies (28,29). In concert with caspase-3 and PARP cleavage, BBR exerted higher activity in those molecular alterations compared with COP, therefore indicating the higher activity of BBR in sensitizing the cells to TRAIL-induced apoptosis. Collectively, these results indicate that BBR may be an attractive adjuvant for TRAIL-targeted therapy for sensitizing cancer cells to TRAIL-induced apoptosis.

### Therapeutic benefit of BBR in combination with anti-DR5 antibody treatment in murine 4T1 breast cancer model

**Figure 3:**
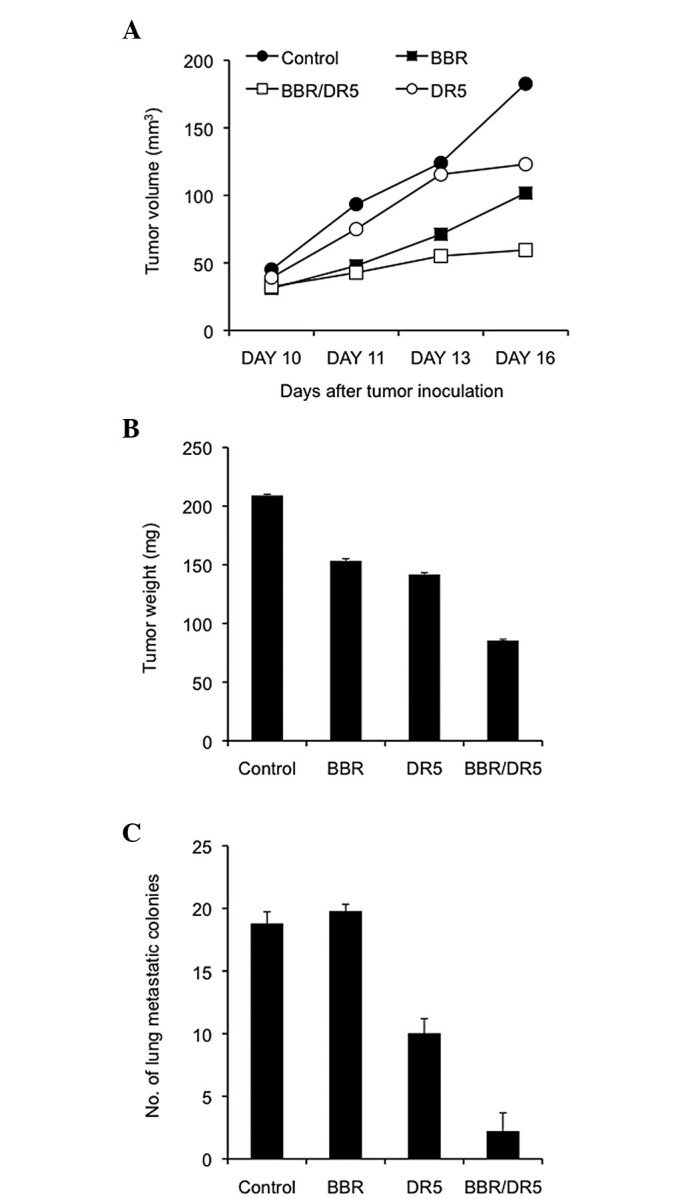
Adjuvant effect of BBR in DR5-targeted therapy in a murine 4T1 breast cancer model. 4T1 cells were inoculated into syngeneic BALB/c female mice that received BBR (100 mg/kg/day) or vehicle by oral administration for one month. Designated mouse groups received agonistic anti-DR5 antibody on days 8 and 13 after tumor inoculation. Tumors were measured on the indicated days and (A) primary tumor growth or (B) tumor weight after removal of the primary tumor on day 16 is shown. (C) The number of lung metastatic colonies was counted following the removal of lung tissue on day 31 (15 days after primary tumor removal). Data are expressed as the mean ± SEM (n=6). BBR, berberine.

To further investigate the therapeutic benefit of BBR in combination with TRAIL-targeted therapy, the adjuvant effect of BBR with an agonistic TRAIL-receptor anti-DR5 antibody (MD5-1) treatment was analyzed in the murine 4T1 breast cancer model. This model is considered to closely resemble the progression of advanced human breast cancer (30). While mice treated with either BBR or anti-DR5 alone showed moderate inhibition of primary tumor growth or tumor weight in the treatment protocol, the combination of BBR and anti-DR5 showed greater inhibition of primary tumor growth rate and tumor weight compared with mice that were treated with either single agent (Fig. 3A and B). Furthermore, the combined treatment also showed a substantial reduction in spontaneous lung metastases of 4T1 tumors (Fig. 3C). Although BBR showed a moderate direct cytotoxic effect on the 4T1 cells *in vitro* (data not shown), the combination of anti-DR5 therapy and BBR effectively inhibited 4T1 tumors at the primary site and further inhibited spontaneous lung metastasis disseminating from the primary tumor.

## Discussion

In the present study, BBR was tested for its ability to sensitize breast cancer to TRAIL-induced apoptosis and its application as an adjuvant for TRAIL-targeted therapy was investigated. BBR not only augmented TRAIL-induced apoptosis in the TRAIL-sensitive MDA-MB-231 breast cancer cells, but also sensitized the TRAIL-resistant MDA-MB-468 breast cancer cells to TRAIL-induced apoptosis. The combination of TRAIL receptor (DR5)-targeted therapy and BBR treatment was demonstrated to effectively inhibit primary tumor growth and metastatic spread in the murine 4T1 breast cancer model. While the anti-metastatic ability of BBR in melanoma has been reported (31), the possibility that the inhibition of lung metastasis is a secondary effect of the inhibition of primary tumor growth may not be excluded. However, the present data clearly suggest that BBR may have therapeutic benefits as an adjuvant in combination with TRAIL/DR5-targeted therapy. Thus, we conclude that BBR may be a promising adjuvant for overcoming TRAIL resistance in TRAIL/DR5-targeted therapy by sensitizing breast cancers to TRAIL/DR5-induced apoptosis.
